# Benzylserine inhibits breast cancer cell growth by disrupting intracellular amino acid homeostasis and triggering amino acid response pathways

**DOI:** 10.1186/s12885-018-4599-8

**Published:** 2018-06-26

**Authors:** Michelle van Geldermalsen, Lake-Ee Quek, Nigel Turner, Natasha Freidman, Angel Pang, Yi Fang Guan, James R. Krycer, Renae Ryan, Qian Wang, Jeff Holst

**Affiliations:** 1 Origins of Cancer Program, Centenary Institute, University of Sydney, Locked Bag 6, Newtown, NSW 2042 Australia; 2 Sydney Medical School, University of Sydney, Sydney, Australia; 3 School of Mathematics and Statistics, University of Sydney, Sydney, Australia; 4 School of Medical Sciences, University of New South Wales, Sydney, Australia; 5 Transporter Biology Group, Discipline of Pharmacology, Faculty of Medicine and Health, University of Sydney, Sydney, Australia; 6 School of Life and Environmental Sciences, University of Sydney, Sydney, Australia; 7 Charles Perkins Centre, University of Sydney, Sydney, Australia

**Keywords:** Amino acids, Metabolism, Triple-negative, Luminal A, Stress response

## Abstract

**Background:**

Cancer cells require increased levels of nutrients such as amino acids to sustain their rapid growth. In particular, leucine and glutamine have been shown to be important for growth and proliferation of some breast cancers, and therefore targeting the primary cell-surface transporters that mediate their uptake, L-type amino acid transporter 1 (LAT1) and alanine, serine, cysteine-preferring transporter 2 (ASCT2), is a potential therapeutic strategy.

**Methods:**

The ASCT2 inhibitor, benzylserine (BenSer), is also able to block LAT1 activity, thus inhibiting both leucine and glutamine uptake. We therefore aimed to investigate the effects of BenSer in breast cancer cell lines to determine whether combined LAT1 and ASCT2 inhibition could inhibit cell growth and proliferation.

**Results:**

BenSer treatment significantly inhibited both leucine and glutamine uptake in MCF-7, HCC1806 and MDA-MB-231 breast cancer cells, causing decreased cell viability and cell cycle progression. These effects were not primarily leucine-mediated, as BenSer was more cytostatic than the LAT family inhibitor, BCH. Oocyte uptake assays with ectopically expressed amino acid transporters identified four additional targets of BenSer, and gas chromatography-mass spectrometry (GCMS) analysis of intracellular amino acid concentrations revealed that this BenSer-mediated inhibition of amino acid uptake was sufficient to disrupt multiple pathways of amino acid metabolism, causing reduced lactate production and activation of an amino acid response (AAR) through activating transcription factor 4 (ATF4).

**Conclusions:**

Together these data showed that BenSer blockade inhibited breast cancer cell growth and viability through disruption of intracellular amino acid homeostasis and inhibition of downstream metabolic and growth pathways.

**Electronic supplementary material:**

The online version of this article (10.1186/s12885-018-4599-8) contains supplementary material, which is available to authorized users.

## Background

Cancer cells require a constant exogenous supply of nutrients to fuel their rapid growth. In recent years, much attention has been given to the importance of amino acids as a substrate for supporting and sustaining tumorigenic proliferation. These amino acids are used for the three critical elements of rapid cell proliferation: biosynthesis of macromolecules, generation of cellular energy, and stimulation of the mTORC1 signalling pathway. In cancer, two particularly crucial amino acids are leucine and glutamine. These two amino acids contribute to the three pathways outlined above but have an additional role in maintaining amino acid balance across the plasma membrane by serving as facultative cotransport or antiport substrates for other amino acids. As a result, cell growth in many cancers is dependent on the availability of leucine and glutamine.

To satisfy these demands, human cancer cells selectively upregulate amino acid transporters to facilitate rapid uptake of amino acid substrates. Leucine uptake is predominantly mediated by the L-type amino acid transporter (LAT) family, a group of four Na^+^-independent transporters (LAT1, SLC7A5; LAT2, SLC7A8; LAT3, SLC43A1; LAT4, SLC43A2) with affinity for branched chain and neutral amino acids [1–4]. Members of the LAT family are significantly upregulated in a number of human malignancies [5], including prostate [6, 7], melanoma [8], and breast. Glutamine transport is largely mediated by alanine, serine, cysteine-preferring transporter 2 (ASCT2; SLC1A5) in multiple cancers, including melanoma [8], non-small cell lung cancer [9, 10], prostate cancer [11], acute myeloid leukaemia [12], multiple myeloma [13], and breast cancer [14, 15]. Other glutamine transporters such as SNAT1 (SLC38A1) and SNAT2 (SLC38A2) have also been shown to play a role in glutamine uptake in triple-negative breast cancer [14], and in osteosarcoma and cervical cancer cells [16]. Together, leucine and glutamine transporters are upregulated in a range of cancers, making them an appealing target for cancer therapy.

As blocking glutamine uptake [14, 15] or inducing leucine deprivation [17] prevents cell growth in triple-negative basal-like breast cancer cells, we hypothesised that dual targeting of glutamine and leucine uptake would be effective in breast cancer, as it is in melanoma [8]. We therefore set out to test whether the published ASCT2 inhibitor, Benzylserine (BenSer), which we have previously shown can also inhibit leucine uptake by LAT1 in melanoma cells [8], would be an effective inhibitor of breast cancer cell growth.

## Methods

### Cell culture

Human breast cancer cell lines HCC1806 and MDA-MB-231 (Catalogue Numbers CRL-2335 and HTB-26 respectively) were purchased from American Type Culture Collection (ATCC), and MCF-7 cell stocks (ATCC Catalogue number HTB-22) were authenticated by STR fingerprinting (CellBank Australia, Westmead, Sydney, NSW, Australia). Cell lines were cultured for up to 30 passages from purchased/authenticated stocks and routinely tested for mycoplasma using PCR detection. MCF-7 cells were grown in MEM medium containing non-essential amino acids (Life Technologies) supplemented with 10% (*v*/*v*) fetal bovine serum (FBS), 2 mM L-glutamine (Life Technologies), 1 mM sodium pyruvate (Life Technologies) and penicillin-streptomycin solution (Sigma-Aldrich, Australia). HCC1806 and MDA-MB-231 cells were grown in RPMI-1640 medium containing L-glutamine and HEPES (Life Technologies) supplemented with 10% (*v*/*v*) fetal bovine serum (FBS; HyClone), 1 mM sodium pyruvate (Life Technologies) and penicillin-streptomycin solution (Life Technologies). Cells were maintained at 37 °C in a fully humidified atmosphere containing 5% CO_2_. Inhibitors were resuspended in H_2_O and diluted 1:10 in media to final concentrations: benzylserine (BenSer, Bachem Swiss), 2-amino-2-norbornanecarboxylic acid (BCH, Sigma-Aldrich), both 10 mM.

### Antibodies

Antibodies used in this study were against α-tubulin (Santa Cruz), pT389-p70S6K, p70S6K, ATF4 (Cell Signalling), and glyceraldehyde-3-phosphate dehydrogenase (GAPDH; Abcam). Horseradish peroxidase-conjugated donkey anti-mouse IgG and anti-rabbit IgG were used as Western blot secondary antibodies (Millipore).

### Uptake assay

To confirm that BenSer inhibits both glutamine and leucine uptake in breast cancer cells, we used a [^3^H]-labelled amino acid uptake assay as described previously [6, 11]. Briefly, cells (1 × 10^5^/well) were incubated at 37 °C in 96-well plates with 0.3 μCi [^3^H]-L-glutamine or [^3^H]-L-leucine (200 nM; Perkin Elmer) in glutamine-free MEM or leucine-free RPMI media (Invitrogen) for 15 min with or without 10 mM BenSer (based on IC_50_ values; Additional file 1: Table S1) or BCH. Cells were transferred to filter paper using a 96-well plate harvester (Wallac PerkinElmer), then the paper was dried, exposed to scintillation fluid and analysed for radiodecay activity using a liquid scintillation counter (PerkinElmer), as described previously [6, 11].

### Cell viability assays

Cells (3 × 10^3^ per well) were plated in 96-well plates and allowed to adhere overnight. Cells were then incubated with or without 10 mM BenSer or BCH for up to 72 h. Media was refreshed every 24 h. Proliferation was measured at days 0, 1, 2, and 3 by the addition of 10 μL MTT solution (5 mg/mL; Millipore) to each well and returning plates to incubation for at least 5 h. Following this, one volume (100 μL) of isopropanol/HCl solution was added to each well and then mixed thoroughly using a multichannel pipette. Absorbance in each well was then read at both 570 nm and 640 nm, the background 570 nm absorbance was subtracted from the 640 nm absorbance, giving the final measurement used for subsequent analysis.

### BrdU incorporation assay

Cells (3 × 10^5^ per well) were plated in 6-well plates and allowed to adhere overnight. Cells were then incubated with or without 10 mM BenSer or BCH for 24 h. BrdU (150 μg/mL) was then added directly to media and incubated for another 2 h. Cells were then collected, fixed, and stained using the BD APC-BrdU flow kit (BD). As per manufacturers’ instructions, the BrdU antibody was diluted 1:50 and nuclei were counter-stained by 7-aminoactinomycin D (7-AAD). Analysis was performed using a BD Fortessa flow cytometer. Data were analysed using FlowJo software (Tree Star Inc.).

### Annexin-V assay

Cells (3 × 10^5^ per well) were plated in 6-well plates, and allowed to adhere overnight, before incubation with or without 10 mM BenSer for 24 h. One well of cells was prepared as a positive control by irradiation in a UV Stratalinker 2400 (Stratagene) with a 400,000 μJ dosage and then incubation in fresh media for 16 h. At the end of the incubation, both adherent and floating cells were collected and resuspended in 100 μl of freshly diluted 1× Annexin V binding buffer (HEPES–buffered PBS supplemented with 2.5 mM calcium chloride) containing anti-Annexin V-APC (BD; diluted 1:100). Samples were then incubated for 30 min in the dark on ice. Following this, propidium iodide (PI) solution (Sigma; 10 μg/mL) was added, and the cells were immediately analysed using a BD Fortessa flow cytometer with data analysis using FlowJo software.

### SDS-PAGE and western blotting

Cells (5 × 10^5^ per well) were plated in 6-well plates and allowed to adhere overnight, before incubation with or without 10 mM BenSer for 6 h. Cells were lysed by the addition of lysis buffer (200 μl) with protease inhibitor Cocktail III (Bioprocessing Biochemical, California) and phosphatase inhibitor (Cell Signalling). Equal protein (as determined by the micro–BCA method; Pierce, IL) was loaded on 4–12% gradient gels (Invitrogen, Australia), electrophoresed and transferred to PVDF membranes using a semi-dry transfer system. Each membrane was blocked with 2.5% (*w*/*v*) BSA in PBS-Tween20 (PBST) and then incubated with the appropriate primary and secondary antibodies. Binding of the secondary HRP-labelled antibodies was detected using enhanced chemiluminescence reagents (Pierce) on a BioRad ChemiDoc (BioRad).

### Gas chromatography-mass spectrometry (GCMS) analysis of intracellular amino acids

Cells were plated in triplicate at a density of 7 × 10^5^ cells/well in 6-well plates and allowed to adhere for 6–8 h. Media was then replaced with 1 mL fresh media containing 100 μL BenSer (final concentration 10 mM) or 100 μL sterile endotoxin-free tissue culture grade water (Sigma) as a vehicle control. After 14 h incubation, intracellular amino acids were extracted by methanol:chloroform extraction. Briefly, medium was removed and the cell monolayer was washed once with 5 mL of ice-cold 0.9% (*w*/*v*) NaCl solution and then rapidly quenched and extracted in 2.5 mL of 50% (*v*/*v*) methanol:water mixture that had been prechilled to − 30 °C. A chlorophenylalanine/norvaline standard mix (Sigma) was added to each well at this step as an internal standard. Cells were scraped into this mixture and then the entire volume was transferred to prechilled Falcon tubes and kept on ice. Each well was then rinsed once with equal volume (2.5 mL) ice-cold UltraPure™ water (ThermoFisher) and this was combined with the first extract. One volume (5 mL) of prechilled chloroform was then added to each tube. The extraction mixes were vortexed vigorously for 10 s and centrifuged at 3200 g for 5 min. The aqueous top phase of each sample was then transferred to a prechilled glass tube, gradually cooled to − 30 °C and then evaporated to dryness without heat using a SpeedVac. Dried samples were promptly derivatised using MTBSTFA and methoxyamine (Sigma), and then analysed by GCMS as described previously [18].

### In silico gene expression analysis

The METABRIC cohort of ~ 2500 clinical breast cancer samples [19, 20] and all breast cancer cell lines included in The Cancer Cell Line Encyclopedia [21] were assessed using cBio Cancer Genomics Portal (www.cbioportal.org). Gene expression (RNA log_2_ expression data) was queried for 9 putative BenSer target transporters: SLC7A5, SLC7A8, SLC3A2, SLC43A1, SLC43A2, SLC1A4, SLC1A5, SLC38A1, and SLC38A2. METABRIC data were additionally sorted on clinical attributes (“Pam50 + Claudin-low subtype”) to assign samples into subtypes. These data were plotted as box-and-whisker plots (whiskers indicating min to max) and analysed using a Kruskal-Wallis test with Dunn’s multiple comparisons correction.

### Oocyte uptake assays

Stage V oocytes were harvested from *Xenopus laevis* as described previously [22]. At least four oocytes per condition were injected with mRNA (SNAT1, SNAT2, ASCT1, ASCT2 or LAT2) and incubated in standard frog Ringer’s solution (ND96: 96 mM NaCl, 2 mM KCl, 1 mM MgCl_2_, 1.8 mM CaCl_2_, 5 mM HEPES, pH 7.5) supplemented with 50 μg/ml gentamycin, 2.5 mM sodium pyruvate, 0.5 mM theophylline at 16–18 °C. Four days after injection, injected and non-injected oocytes were pre-incubated with 10 mM BenSer for five minutes at room temperature, incubated with [^3^H]-labelled glutamine (SNAT1, SNAT2 and ASCT2), serine (ASCT1) or leucine (LAT2) and 10 mM BenSer at room temperature for 10 mins (30 min for LAT2), and then washed three times in ice cold uptake solution. Predicted EC_20_ values from electrophysiology were SNAT1 (35 μM), SNAT2 (145 μM), ASCT1 (22 μM) and ASCT2 (18 μM). For LAT2, 1 mM was used in the experiment. For SNAT and ASCT transporters, the uptake solution was ND96. For LAT2 the uptake solution was a sodium-free buffer identical to ND96, except that sodium was replaced with the cation, choline. Washing was followed by lysis in 1 M NaOH and 1% SDS. [^3^H]-L-substrate uptake was measured by scintillation counting using a Trilux beta counter (Perkin Elmer). A separate group of control cells were subjected to the same uptake procedures, in the absence of BenSer. All experiments were performed in quadruplicate and repeated using oocytes harvested from at least two different animals.

### Seahorse Mito stress test assay

All wells of the Seahorse XF^e^ 96-well plate were treated with poly-D-lysine and then cells (2 × 10^4^ cells/well) were plated and allowed to adhere overnight. The Seahorse XF^e^ sensor cartridge was hydrated overnight according to manufacturer’s instructions. The next day, the cell culture media in the XF^e^ 96-well plate was removed and each well was washed once with Seahorse XF Assay Medium. Fresh Assay Medium (180 μL) containing either BenSer (10 mM), BCH (10 mM) or vehicle control (sterile endotoxin-free water; Sigma) was added to each well. The XF^e^ 96-well plate was then incubated for 1 h at 37 °C in a non-CO_2_ incubator, as per the manufacturer’s instructions. The overnight pre-hydrated sensor cartridge was then loaded with the mitochondrial inhibitors oligomycin, FCCP, and rotenone and antimycin A, which were provided in the Mito Stress Test kit and diluted just prior to use according to manufacturer’s instructions. These inhibitors were delivered sequentially from ports A (oligomycin; 1.3 μM), B (FCCP; MCF-7 0.25 μM; HCC1806 and MDA-MB-231 0.5 μM), and C (rotenone 0.5 μM and antimycin A 0.5 μM) in all wells, to measure ATP–linked respiration, maximal respiration, and non-mitochondrial respiration, respectively.

The loaded sensor cartridge was then calibrated in the Seahorse XF^e^96 machine according to manufacturer’s instructions, before being loaded into the XF^e^ 96-well plate for commencement of the Mito Stress Test Assay. Oxygen consumption rate (OCR) and extracellular acidification rate (ECAR) in each well was measured at 6.5 min intervals for 130 min. These measurements captured three baseline measurements (“basal respiration”), four measurements post-oligomycin injection (“ATP-linked respiration”), four measurements post-FCCP injection (“maximal respiration”), and four measurements post-rotenone/antimycin A injection (“non-mitochondrial respiration”). Proton leak and spare respiratory capacity were calculated from the OCR measurements according to manufacturer’s instructions.

## Results

### BenSer inhibits leucine and glutamine uptake in breast cancer cells

**Fig. 1:**
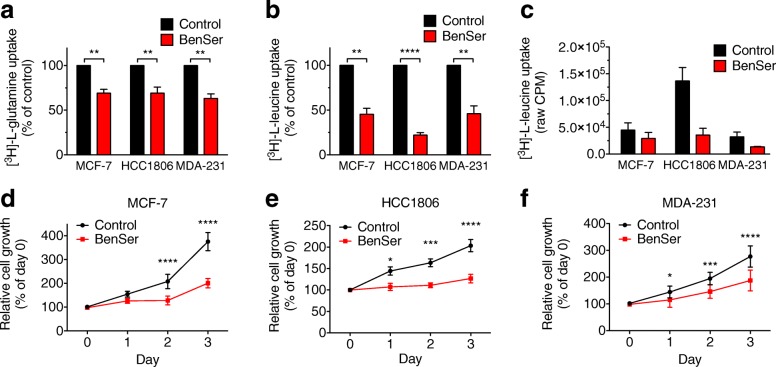
BenSer inhibits breast cancer cell growth by blocking leucine and glutamine uptake. Glutamine (**a**) and leucine (**b**) uptake over 15 min were measured in MCF-7, HCC1806 and MDA-MB-231 (MDA-231) cells in the presence or absence of 10 mM BenSer. **c**, data from (**b**) showing raw counts per minute (CPM). **d**-**f**, relative cell viability measured by MTT assay in MCF-7 (**d**), HCC1806 (**e**), and MDA-231 (**f**) cells cultured for 3 days in the presence or absence of 10 mM BenSer. Data represent mean ± SEM of at least three independent experiments. *p < 0.05, **p < 0.01, ***p < 0.001, ****p < 0.0001; unpaired student’s t-test (**a**-**b**), 2-way ANOVA (**d**-**f**)

Using three different breast cancer cell lines: estrogen-receptor (ER)-positive, Luminal A MCF-7 cells, triple-negative basal-like HCC1806 cells, and triple-negative claudin-low MDA-MB-231 cells, to represent a variety of breast cancer subtypes, we showed that treatment with BenSer reduced glutamine uptake to ~ 65% of control across all three cell lines (Fig. 1a), while leucine uptake was inhibited more strongly to ~ 45% (MCF-7 and MDA-MB-231) and 22% (HCC1806) of control (Fig. 1b). Previous data have shown that total glutamine uptake in these three cell lines is HCC1806 > MDA-MB-231 > MCF-7 (CPM > CPM > CPM; [15]). Despite these variations in glutamine uptake, the % inhibition after BenSer was similar for all three cell lines. Analysis of total leucine uptake again showed the highest level in HCC1806, with much lower levels in MCF-7 and MDA-MB-231 cells (Fig. 1c). Interestingly, despite this high leucine uptake in HCC1806 cells, BenSer had the largest effect on leucine uptake in this cell line. As this uptake assay is performed over a short time course (15 min), these data suggested that BenSer was able to acutely inhibit both glutamine and leucine uptake in breast cancer cells.

### BenSer inhibits breast cancer cell growth

To determine the effect of BenSer on breast cancer cell growth, cells were cultured in the presence of 10 mM BenSer for 3 days and cell viability was indirectly measured every 24 h using an MTT assay. BenSer treatment significantly reduced cell viability in all three cell lines, regardless of their subtype or proliferation rate (Fig. 1d-f). Inhibitory effects were observed within the first 48 h in all cell lines. We next set out to determine whether cell cycle blockade or apoptosis contributed to the reductions in cell growth observed with MTT.

**Fig. 2:**
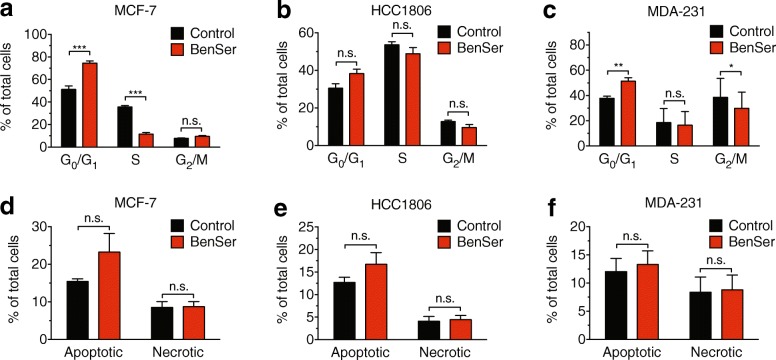
BenSer treatment reduces cell cycle progression with minimal effects on apoptosis. Cell cycle progression measured by BrdU incorporation in MCF-7 (**a**), HCC1806 (**b**) and MDA-MB-231 (MDA-231; **c**) cells cultured in the presence or absence of 10 mM BenSer for 24 h. Annexin-V staining in MCF-7 (**d**), HCC1806 (**e**) and MDA-MB-231 (**f**) cells cultured in the presence or absence of 10 mM BenSer for 24 h. Data represent mean ± SEM of at least three independent experiments. *p < 0.05, **p < 0.01, ***p < 0.001, ****p < 0.0001; 2-way ANOVA

We first used BrdU incorporation and 7-AAD staining to analyse cell cycle phase. Each cell line showed distinct differences in baseline cell cycle profiles, with MCF-7 cells being mainly in G_0_/G_1_, HCC1806 cells in S phase, and MDA-MB-231 cells in G_2_/M phase. Despite these differences in baseline cell cycle profile, BenSer treatment reduced cell cycle progression, resulting in a 10–20% increase in cells accumulated at G_0_/G_1_ phase (Fig. 2a-c), although this effect was only significant in MCF-7 cells and MDA-MB-231 cells, suggesting that processes other than cell cycle arrest also contribute to reduced cell growth. In MCF-7 and HCC1806 cells, the increase of cells in G_0_/G_1_ was accompanied by decreases in S phase, and in MDA-MB-231 cells, a decrease in G_2_/M phase. We next examined apoptosis using flow cytometry to detect levels of “flipped” Annexin-V in the plasma membrane, combined with PI to measure cell permeability. Treatment with BenSer for 24 h did not significantly increase the number of apoptotic (Ann^+^/PI^−^ and Ann^+^/PI^+^) cells in MCF-7, HCC1806, and MDA-MB-231 cells (Fig. 2d-f), suggesting that the effects of BenSer treatment were predominantly cytostatic. These data show a broad applicability of BenSer treatment across different breast cancer subtypes and proliferation rates, in contrast to previous data showing ASCT2 inhibition alone affects highly proliferative triple-negative cancer cells (HCC1806, MDA-MB-231), but not Luminal A breast cancer cell lines such as MCF-7 [15].

**Fig. 3:**
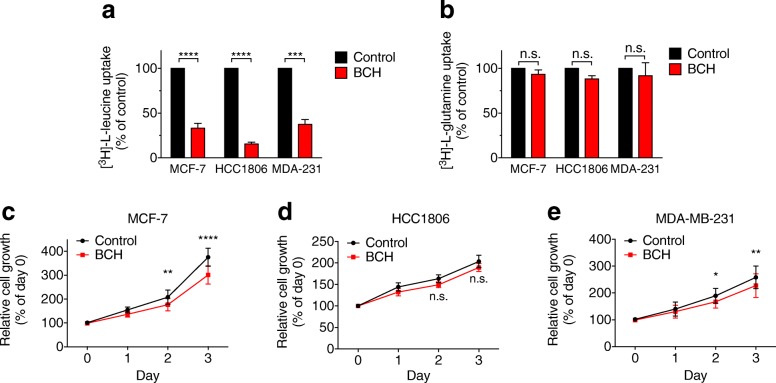
Inhibition of leucine uptake alone does not recapitulate the effects of BenSer. Leucine (**a**) and glutamine (**b**) uptake over 15 min were measured in MCF-7, HCC1806 and MDA-MB-231 (MDA-231) cells in the presence or absence of 10 mM BCH. Relative cell viability measured by MTT assay in MCF-7 (**c**), HCC1806 (**d**), and MDA-MB-231 (**e**) cells cultured for 3 days in the presence or absence of 10 mM BCH. Data represent mean ± SEM of at least three independent experiments. *p < 0.05, **p < 0.01, ***p < 0.001, ****p < 0.0001; unpaired student’s t-test (**a**-**b**), 2-way ANOVA (**c**-**e**)

To investigate this further, we used the LAT family inhibitor, BCH, to determine whether the growth inhibitory effects of BenSer were leucine-mediated. BCH treatment potently blocked [^3^H]-L-leucine uptake to a similar extent in all three cell lines (Fig. 3a), without any effect on [^3^H]-L-glutamine uptake (Fig. 3b). However, unlike BenSer, BCH treatment caused only a modest but significant (~ 10%) reduction in cell viability in MCF-7 and MDA-MB-231 cells (Fig. 3c, e), and no significant effect on HCC1806 cell viability (Fig. 3d). These data suggest that the dual targeting of leucine and glutamine transporters by BenSer may have an additive or synergistic effect, and that reduced uptake of both amino acids is required for optimal inhibition of cell growth. Interestingly, however, combined treatment of MCF-7 and HCC1806 cells with both a leucine uptake inhibitor (BCH) and a glutamine uptake inhibitor (L-γ-glutamyl-p-nitroanilide; GPNA), did not recapitulate the effects of BenSer treatment (Additional file 2: Figure S1A-B), suggesting that BenSer may block uptake of leucine and glutamine by acting on other additional amino acid transporters.

### BenSer directly inhibits other targets that are expressed in breast cancer cell lines and patient samples

**Fig. 4:**
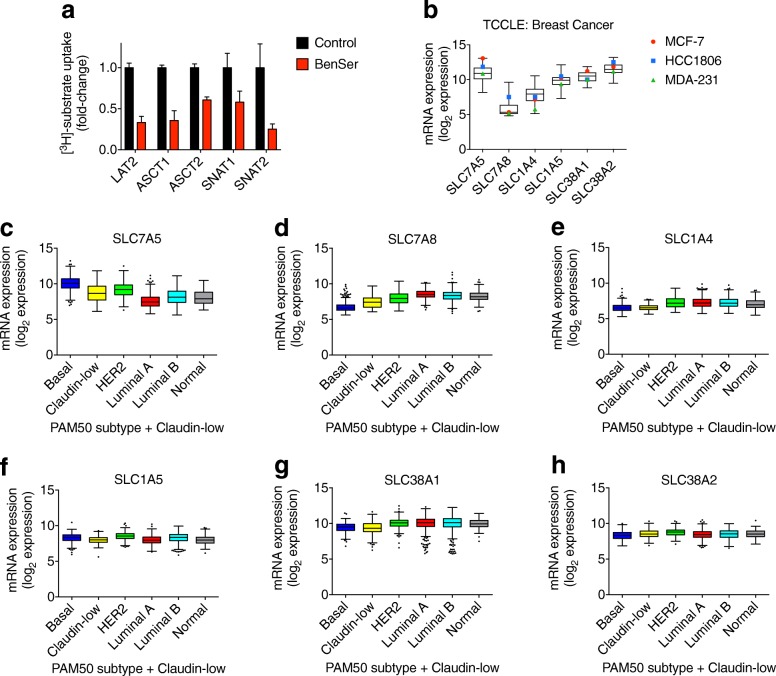
Novel BenSer targets are expressed in breast cancer. **a**, [^3^H]-substrate uptake was assessed in oocytes expressing LAT2/SLC7A8 and heavy chain SLC3A2, ASCT1/SLC1A4, ASCT2/SLC1A5, SNAT1/SLC38A1, or SNAT2/SLC38A2 in the presence or absence of 10 mM BenSer. Data from one representative batch of oocytes are presented. Each datapoint represents the mean ± SEM values (*n* ≥ 4) for the difference between the mean uptake by ‘*n*’ injected oocytes and the mean uptake by ‘*n*’ uninjected oocytes. The variance of this difference was calculated using Gauss’ law of error propagation. Data were normalised to the control condition (uptake in the absence of BenSer). **b**, gene expression (mRNA log_2_ values) of LAT transporters (SLC7A5, SLC7A8, SLC43A1, SLC43A2), LAT common heavy chain (SLC3A2) and glutamine transporters (SNATs: SLC38A1, SLC38A2; ASCT2/SLC1A5, ASCT1/SLC1A4) was analysed in all breast cancer cell lines (*n* = 55) included in The Cancer Cell Line Encyclopedia (TCCLE). Grouped data are plotted as box-and-whisker plots (max to min), with log_2_ mRNA expression in MCF-7 (red), HCC1806 (blue) and MDA-MB-231 (MDA-231; green) cells overlaid as individual data points. **c**-**h**, mRNA expression (log_2_ values) of SLC7A5 (**c**), SLC7A8 (**d**), SLC1A5 (**e**), SLC1A4 (**f**) SLC38A1 (**g**), and SLC38A2 (**h**) in the METABRIC dataset (*n* = 2509). Data were grouped into the “PAM50 + Claudin-low” subtypes based on clinical attribute data retrieved from www.cbioportal.org/ and are plotted as box-and-whisker plots (Tukey)

In light of these data, we next examined the ability of BenSer to directly inhibit the uptake activity of other putative targets, such as additional LAT transporters (LAT2/SLC7A8), glutamine transporters (SNATs; SLC38A1, SLC38A2) and other structurally similar amino acid transporters (ASCT1/SLC1A4), as well as confirming its known inhibition of ASCT2/SLC1A5. Using a *Xenopus laevis* oocyte expression system, the substrate uptake activity of LAT2 (SLC7A8; co-expressed with its heterodimeric heavy chain, SLC3A2), ASCT1 (SLC1A4), ASCT2 (SLC1A5), SNAT1 (SLC38A1) and SNAT2 (SLC38A2) was inhibited in the presence of BenSer (Fig. 4a), as we have previously shown for LAT1 [8]. These data suggest that the inhibition of breast cancer cell growth caused by BenSer treatment is mediated in part by direct inhibition of multiple amino acid transporters, and not just by inhibition of LAT1 and ASCT2.

Although not normally expressed at high levels in breast tissue, we and others have reported that breast cancers [14, 23–25] and breast cancer cell lines [15, 26, 27] express high levels of the known BenSer targets, LAT1 and ASCT2, but little is known about the expression of these other novel BenSer targets in breast cancer [28–32]. Using cBioPortal, we assessed the expression of these transporters in the METABRIC cohort of ~ 2500 clinical breast cancer samples [19, 20] and all breast cancer cell lines included in The Cancer Cell Line Encyclopedia [21]. These analyses confirmed aberrantly high expression of LAT1 (SLC7A5) and its heterodimeric heavy chain, SLC3A2, in cell lines (Fig. 4b, Additional file 2: Figure S2A) and clinical samples (Fig. 4c, Additional file 2: Figure S2B), with significantly higher expression in more proliferative breast cancer subtypes (Fig. 4c, Additional file 2: Figure S2C, Additional file 1: Table S2; basal, claudin-low, HER2; *p* < 0.0001, Kruskal-Wallis test). These analyses also showed high expression of the other novel BenSer target transporters (Fig. 4d-h, and two other LAT family transporters (LAT3/SLC43A1, LAT4/SLC43A2; Additional file 2: Figure S2C-D), but with little difference in expression across genetic subtypes (“PAM50 classification plus claudin-low”), suggesting that upregulation of these transporters occurs non-specifically in breast cancer and that therapies designed to target these pathways could have broad efficacy across clinical subgroups.

### BenSer treatment affects multiple pathways of intracellular amino acid metabolism and activates AAR signalling

**Fig. 5:**
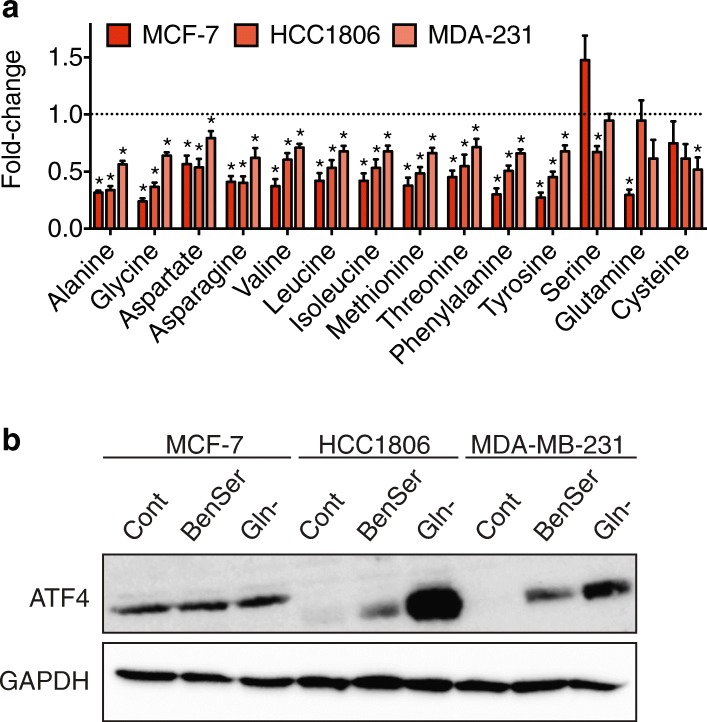
BenSer reduces intracellular amino acid concentrations and activates amino acid response signalling pathways. **a**, intracellular levels of alanine, glycine, asparagine, aspartate, valine, leucine, isoleucine, methionine, threonine, phenylalanine, tyrosine, serine, glutamine and cysteine were measured in MCF-7, HCC1806 and MDA-MB-231 (MDA-231) cells after 14 h incubation in the presence or absence of 10 mM BenSer. Data are normalised to cellular protein content and expressed as a fold-change compared to Control. Data represent mean ± SEM of two independent experiments performed in triplicate. *p < 0.05, **p < 0.01, ***p < 0.001, ****p < 0.0001; unpaired student’s t-test. **b**, levels of ATF4 (49 kDa) protein detected by Western blotting in MCF-7, HCC1806 and MDA-MB-231 cells cultured in the presence or absence of 10 mM BenSer for 6 h. Cells cultured in glutamine-free (Gln-) media were included in all blots as a positive control and GAPDH (37 kDa) was used as a loading control. Data in (**b**) are representative blots from at least three independent experiments

Due to BenSer’s ability to bind and block multiple amino acid transporters, we next examined the effect of BenSer treatment (14 h) on intracellular amino acid concentrations using GCMS (Fig. 5a). BenSer treatment significantly reduced the intracellular concentrations of the small neutral amino acids, alanine, glycine, and asparagine, by ~ 50% in all three cell lines. Intracellular aspartate, the deamination product of asparagine, was similarly reduced across all three cell lines but to a lesser extent (0.3-fold reduction). An additional seven neutral amino acids (valine, leucine, isoleucine, methionine, threonine, phenylalanine, tyrosine) showed reduced intracellular concentrations in all three cell lines. For all of these seven amino acids, the fold-change decrease was greater in MCF-7 cells than HCC1806 or MDA-MB-231 cells, indicating a stronger effect in MCF-7 cells. In contrast, levels of serine, glutamine, and cysteine were each reduced in only one cell line. Levels of the remaining 4 amino acids (glutamate, lysine, arginine, tryptophan) were not significantly altered in any cell line (Additional file 2: Figure S3). Proline and histidine levels were not measured in these experiments. These data show that BenSer treatment affects the intracellular concentration of at least 14 amino acids, and that these are not limited to LAT1 or ASCT2 substrates, indicating that BenSer treatment can disrupt multiple amino acid uptake and metabolism pathways, and suggesting this is a possible mechanism by which it exerts its growth inhibitory effects.

We next set out to determine whether the altered intracellular amino acid concentrations caused by BenSer treatment activates amino acid response (AAR) pathways, a critical sensor of amino acid levels. GCN2 is an important mediator of the AAR pathway, where it senses uncharged tRNA abundance – increased when intracellular amino acid availability is low – and activates signal transduction pathways through phosphorylation of eIF2α. A major outcome of this signalling pathway is increased expression of ATF4 [33], mediated by upregulated translation in response to nutrient starvation [34]. We performed Western blotting for ATF4 protein in breast cancer cells treated with BenSer for 6 h to determine whether BenSer-induced disruption of amino acid homeostasis was sufficient to induce the AAR pathway. These blots showed increased expression of ATF4 in HCC1806 and MDA-MB-231 only (Fig. 5b), indicating activation of the AAR in these cells. Interestingly, MCF-7 cells showed basal ATF4 expression, suggesting AAR stress pathways are constitutively activated in this cell line. These differences may be due to the subtype-specific alterations in intracellular amino acid levels seen in response to BenSer treatment (Fig. 5a), or may simply reflect different subtype-specific reliance on particular amino acids as a substrate for cell growth and metabolism, just as we have previously shown for glutamine [15].

### BenSer treatment affects glycolytic but not oxidative metabolism

**Fig. 6:**
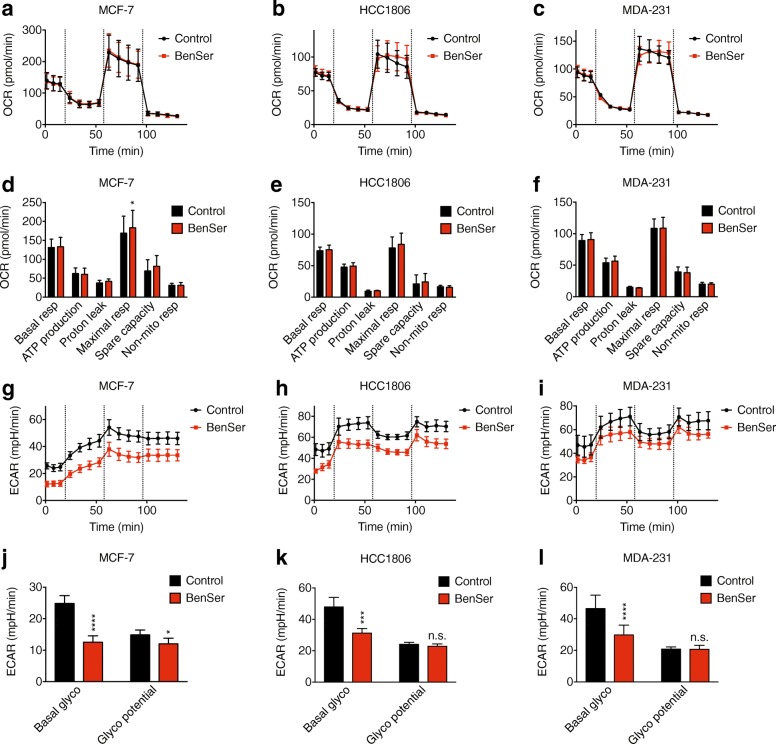
BenSer treatment reduces ECAR. Oxygen consumption rate (OCR; **a**-**f**) and extracellular acidification rate (ECAR; **g**-**l**) in MCF-7, HCC1806 and MDA-MB-231 (MDA-231) cells treated in the presence or absence of 10 mM BenSer were assessed using a Seahorse Mito Stress Test. Data represent mean ± SEM of 3–4 independent experiments performed in triplicate. *p < 0.05, **p < 0.01, ***p < 0.001, ****p < 0.0001; unpaired student’s t-test

As amino acids can be used to fuel oxidative metabolism, we next assessed the oxygen consumption rate (OCR) after treatment with BenSer. BenSer treatment did not affect oxidative metabolism (Fig. 6a-f) in any of the cell lines, except in MCF-7 cells where there was a small but significant increase in maximal respiratory capacity (Fig. 6d). Next, we measured the extracellular acidification rate (ECAR) to examine the effect of BenSer on basal and glycolytic potential (difference between oligomycin-treatment and basal treatment), as cancer cells can compensate for decreased OCR by increasing ECAR to maintain steady ATP production. BenSer significantly reduced basal ECAR in all three cell lines (Fig. 6g-l), and also caused a small reduction in glycolytic potential of MCF-7 cells only (Fig. 6j). This suggested that disrupted amino acid homeostasis caused by BenSer induces a compensatory shift away from lactate-producing glycolysis in order to maintain oxidative phosphorylation.

To confirm that this was not simply due to depletion of leucine, but rather an effect of disrupted amino acid homeostasis specific to BenSer treatment, we also examined OCR and ECAR after BCH treatment. In contrast to BenSer, BCH treatment resulted in a significant decrease in basal OCR and spare respiratory capacity (Additional file 2: Figure S4A-F) in all 3 cell lines, but had no effect on ECAR (Additional file 2: Figure S4G-L). This indicated that with sole inhibition of leucine uptake (BCH), cells do not compensate for reduced OCR by increasing ECAR, whereas with reduced intracellular levels of multiple amino acids (BenSer), ECAR is reduced instead of OCR (Additional file 2: Figure S4M-O). This clearly demonstrates different and specific bioenergetic responses to the unique metabolic stresses induced by inhibition of different transporters.

## Discussion

Intracellular amino acid concentrations are regulated by interconnected systems of exchange, influx and efflux, all mediated by specific cell surface transporters. Additional regulation of transporter function by transcriptional, translational, and allosteric mechanisms, is overlaid onto these processes, creating a complex regulatory network. We and others have shown that altering the levels of only a single amino acid [17, 35] or a single transporter [6, 15] is sufficient to impair or prevent cancer cell growth. There also appears to be cell type-specific tropism for particular amino acids and/or their transporters, as the key therapeutic targets identified thus far differ markedly across malignancies; or indeed, of note in breast cancer, within subtypes of the same cancer. For example, Luminal A breast cancer cells are resistant to ASCT2 inhibition while triple-negative breast cancer cells are exquisitely sensitive [15]. However, due to the dynamic nature of amino acid transport, and the primacy of leucine and glutamine in maintaining these processes, we proposed that modulating the levels of these two amino acids may be sufficient to disrupt whole cell amino acid balance across multiple breast cancer subtypes, as it has been shown in melanoma [8].

This study used the amino acid analogue, BenSer, for testing the efficacy of dual leucine-glutamine uptake inhibition through inhibition of LAT1 and ASCT2. BenSer was first characterised as a specific ASCT2 inhibitor in 2004 [36] and has since been used as a competitive inhibitor of small, neutral amino acid transport in cancer cell lines [8, 11]. However, to date, the exact mechanism by which it exerts its anti-growth action remains largely unknown; for example, we have shown using *Xenopus laevis* oocyte uptake assays that BenSer can also directly inhibit LAT1 [8], and now show inhibitory activity against LAT2, ASCT1, SNAT1 and SNAT2 (Fig. 4a), confirming previous reports at higher doses [16]. In this study, we have also shown that BenSer inhibits breast cancer cell growth by preventing uptake of amino acids by these transporters, thus disrupting intracellular amino acid homeostasis, glycolysis, and triggering AAR pathways.

Notably, 14 h BenSer treatment reduced intracellular glutamine levels in MCF-7 cells only, despite significantly inhibiting uptake in all three cell lines in a 15 min [^3^H]-glutamine uptake assay. This suggests that breast cancer cells may display some metabolic flexibility when amino acid homeostasis is disrupted, causing cells to prioritise the regeneration of particular amino acid pools within the cell as an adaptive mechanism. Both glutamine and glutamate play a critical role within the cell by acting as donors and acceptors of nitrogen for biosynthetic reactions, and thus replenishment of this pool is critical for sustaining multiple cellular metabolic processes. Breast cancer cells express high levels of glutamine metabolism-related enzymes, including glutamine synthetase [15, 37], which permits the synthesis of glutamine from glutamate and ammonia.

Furthermore, glutamine/glutamate/αketoglutarate flux provides fuel for TCA cycle in many cancer cells. We therefore assessed OCR in each cell line after BenSer, but found there were no differences in basal or maximal OCR, suggesting BenSer treatment may force cells to prioritise other substrates, such as glucose or fatty acids, into TCA cycle to replace anaplerotic intermediates derived from amino acid substrates. While BenSer did not affect OCR, it caused a major shift in ECAR, suggesting a reduction in lactate production. This may indicate rerouting of metabolism away from glycolysis to provide carbons for serine/glycine metabolism, or into TCA cycle in an attempt to compensate for the loss of other amino acids. Alternatively, reduced ECAR may simply reflect the reduced growth rate caused by BenSer treatment (Figs. 1 and 2), indicating a paired inhibition of both glycolytic and biosynthetic pathways in the context of reduced intracellular amino acids; for example, as a result of reduced flux through the pentose phosphate pathway due to increased glycolytic flux to replenish TCA, occurring alongside reduced protein biosynthesis in response to depleted amino acids. Further studies encompassing global metabolomics analysis are needed to delineate the mechanisms of these potential adaptive feedback loops.

Interestingly, BCH treatment significantly reduced basal and maximal OCR (Additional file 2: Figure S4), suggesting the changes in amino acid balance through LAT inhibition may negatively affect OCR without any compensatory mechanisms triggered (no change in ECAR), perhaps through central pro-growth signalling axes such as mTORC1 [38]. Unlike glutamine, leucine is an essential amino acid, and therefore cannot be synthesised by enzymatic reactions within the cell. It was therefore not surprising that leucine levels remained reduced at 14 h, and this may also suggest that leucine-dependent metabolic processes are less critical to cell survival than those that require glutamine or glutamate, enabling cell growth to continue when there is residual minimal leucine (Fig. 3) despite being inhibited by complete leucine deprivation [17].

Although the growth of all breast cancer cell lines was inhibited by BenSer treatment, there were some cell line-specific effects. Overall, MCF-7 cells were the most sensitive to BenSer treatment, showing the strongest inhibition of cell growth (Figs. 1d-f, 2a-c), reduction of intracellular amino acid concentrations (Fig. 5a), and with a background of basally activated ATF4 (Fig. 5b), all suggesting a greater reliance on amino acids to drive TCA (Fig. 6). In contrast, the claudin-low triple-negative cell line, MDA-MB-231, showed the mildest effects when treated with BenSer. As MDA-MB-231 cells also express a gene signature associated with epithelial-to-mesenchymal transition (EMT) processes involved in metastasis [39], this may suggest altered amino acid requirements during metastatic progression of breast cancer cells, as shown by others recently [40]. Notably we did not see a significant difference in transporter gene expression in MDA-MB-231 cells (Fig. 4b) or claudin-low tumours from the METABRIC cohort (Fig. 4c-h), indicating that expression levels do not necessarily correlate with the importance of a transporter, as we have shown previously for ASCT2 [15]. This highlights the need for future studies to identify an appropriate biomarker that can prospectively stratify individual breast tumours on their responsiveness to dual leucine-glutamine deprivation strategies.

As a structural analogue of ASCT2 substrates, BenSer has binding affinity in the high micromolar range [36] but usually requires millimolar doses to inhibit transport in human cell lines, likely due to competition with high concentrations of amino acids in the culture media [8]. Although we showed that BenSer was efficacious in reducing breast cancer cell growth in vitro, at these concentrations, the drug is well outside the appropriate range for clinical translation. Therefore, higher affinity compounds with greater selectivity would need to developed to enable testing at clinically relevant doses. Additionally, future in vitro studies using BenSer as a specific ASCT2 inhibitor should interpret results with caution, as our data from oocyte uptake assays indicated off-target binding to other transporters (SNAT1, SNAT2, ASCT1, LAT2; Fig. 4a) in the millimolar effective dose range, which we propose contributes to its mechanism of action. However, it remains unclear whether all or just some of these transporters are required to mediate the growth-inhibitory effect of BenSer.

Recent data have shown how ATF4, MYC and mTORC1 stress-response pathways may converge in cancers that are reliant on glutamine [41–45], such as triple-negative breast cancer, where oncogenic MYC is a suspected driver of glutamine-reliance [46]. This may suggest a unique therapeutic sensitivity to AAR pathway disruption in this currently underserved patient population. However, even breast cancers that are not MYC-driven appear to rely on intact AAR pathways to drive adaptive cancer cell survival; for example, leucine deprivation in glutamine-independent luminal breast cancer cells inhibits growth via GCN2-mediated signalling [17]. This suggests that a therapy targeting AAR pathways (e.g GCN2 inhibition) may have synergy with amino acid uptake inhibitors, thus extending their efficacy to a broader patient population.

This study has provided proof of principle data to support the biological feasibility of dual leucine-glutamine restriction for inhibiting breast cancer cell growth. BenSer treatment restricted cell growth in three different breast cancer cell lines, each representative of a different clinical subtype, growth rate, and metastatic potential. Further dose-response studies are needed to clarify the minimum restriction required to slow cell growth. Previous data have shown that the intensity and duration of stress conditions dictate the response driven by AAR pathway activation; low levels of cell stress may contribute to adaptive resistance mechanisms, whereas high levels promote apoptosis [47, 48]. Like inhibitors of mTORC1 signalling, therapies targeting this axis have the potential to either promote or prevent tumour cell survival, and therefore future studies should focus on delineating these pharmacodynamic effects. This is especially pertinent in light of the cell line-specific stress responses that we observed in response to BenSer (Figs. 5b, 6), indicating that the same treatment may stimulate different adaptive responses across breast cancer subtypes.

## Conclusions

Amino acids are critical for sustaining cancer cell metabolism, with leucine and glutamine playing key roles in promoting cell growth, sustaining TCA cycle, and maintaining amino acid homeostasis. Dual inhibition of leucine and glutamine uptake by the pharmacological inhibitor, BenSer, was able to reduce growth of breast cancer cells and limit cell cycle progression. BenSer treatment also reduced intracellular concentrations of multiple amino acids, indicating disrupted amino acid homeostasis. Oocyte uptake assays demonstrated that this was caused at least in part by direct inhibition of additional BenSer targets, LAT2, ASCT1, SNAT1, and SNAT2. Reduced lactate production after BenSer treatment suggested dynamic metabolic responses to amino acid transporter inhibition, and this was accompanied by activation of AAR pathways as an adaptive stress response. Notably, these effects were observed in three cell lines representative of different breast cancer subtypes, indicating that the effects of BenSer are not subtype-restricted but may have broad applicability.

## Additional files

Additional file 1: **Table S1.** Summary of [^3^H]-L-glutamine and [^3^H]-L-leucine uptake IC_50_ values for breast cancer cells treated with varying concentrations of BenSer. **Table S2.** Summary of Kruskal-Wallis tests with Dunn’s multiple comparisons correction on inter-subtype comparisons of gene expression (mRNA log_2_ values) of putative BenSer targets SLC7A5, SLC3A2, SLC7A8, SLC1A5, SLC1A4, SLC38A1, and SLC38A2 in the METABRIC dataset (*n* = 2509). Data were grouped into the “PAM50 + Claudin-low” subtypes based on clinical attribute data retrieved from www.cbioportal.org/. *p < 0.05, **p < 0.01, ***p < 0.001, ****p < 0.0001, ns = not significant. (PDF 308 kb)

Additional file 2: **Figure S1.** Combined LAT1 and ASCT2 inhibition does not recapitulate the effects of BenSer. Relative cell viability measured by MTT assay in MCF-7 (A) and HCC1806 (B) cells cultured for 3 days in the presence or absence of 10 mM BCH and 1 mM GPNA. Data represent mean ± SEM of at least three independent experiments. *p < 0.05, **p < 0.01, ***p < 0.001, ****p < 0.0001; 2-way ANOVA. **Figure S2.** Expression of putative BenSer targets in breast cancer cell lines and patient samples**.** A, gene expression (mRNA log_2_ values) of LAT common heavy chain (SLC3A2) and LAT transporters (LAT3/SLC43A1, LAT4/SLC43A2), was analysed in all breast cancer cell lines (*n* = 55) included in The Cancer Cell Line Encyclopedia (TCCLE). Grouped data are plotted as box-and-whisker plots (max to min), with log_2_ mRNA expression in MCF-7 (red), HCC1806 (blue) and MDA-MB-231 (MDA-231) cells overlaid as individual data points. B, mRNA expression (log_2_ values) of SLC3A2 (B), SLC43A1 (C) and SLC43A2 (D), in the METABRIC dataset (*n* = 2509). Data were grouped into the “PAM50 + Claudin-low” subtypes based on clinical attribute data retrieved from www.cbioportal.org/ and are plotted as box-and-whisker plots (Tukey). **Figure S3.** Intracellular levels of amino acids that are not affected by BenSer treatment. Intracellular levels of glutamate (A), lysine (B), arginine (C), and tryptophan (D) measured in MCF-7, HCC1806 and MDA-MB-231 (MDA231) cells after 14 h incubation in the presence or absence of 10 mM BenSer. Data are normalised to cellular protein content and expressed as a fold-change compared to Control. Data represent mean ± SEM of two independent experiments performed in triplicate. For (D), *p*-value shown was calculated with unpaired student’s t-test. **Figure S4.** BCH treatment reduces OCR. Oxygen consumption rate (OCR; A-F) and extracellular acidification rate (ECAR; G-L) in MCF-7, HCC1806 and MDA-MB-231 cells treated in the presence or absence of 10 mM BCH were assessed using a Seahorse Mito Stress Test. Data represent mean ± SEM of 3–4 independent experiments performed in triplicate. Average basal OCR and ECAR measurements were then plotted against each other for each treatment condition (M-O). *p < 0.05, **p < 0.01, ***p < 0.001, ****p < 0.0001; unpaired student’s t-test. (PDF 2866 kb)

## Abbreviations

7-AAD: 7-Aminoactinomycin D
AAR: Amino acid response
ANOVA: Analysis of variance
APC: Allophycocyanin
ASCT: Alanine, serine, cysteine-preferring transporter
ATF4: Activating transcription factor 4
ATP: Adenosine triphosphate
BCA: Bicinchoninic acid assay
BCH: 2-Amino-2-norbornanecarboxylic acid
BenSer: Benzylserine
BrdU: 5-Bromo-2′-deoxyuridine
CPM: Counts per minute
ECAR: Extracellular acidification rate
EMT: Epithelial-to-mesenchymal transition
ER: Estrogen receptor
FBS: Foetal bovine serum
FCCP: Carbonyl cyanide p-trifluoromethoxy-phenylhydrazone
GAPDH: Glyceraldehyde-3-phosphate dehydrogenase
GCMS: Gas chromatography-coupled mass spectrometry
GCN2: General control nonderepressible 2
Ig: Immunoglobulin
LAT: L-type amino acid transporter
METABRIC: Molecular Taxonomy of Breast Cancer International Consortium
MTBSTFA: N-tert-Butyldimethylsilyl-N-methyltrifluoroacetamide
mTORC1: Mammalian target of rapamycin complex 1
MTT: 3-(4,5-dimethylthiazol-2-yl)-2,5-diphenyltetrazolium bromide
OCR: Oxygen consumption rate
PBST: Phosphate buffered saline-Tween20
PI: Propidium iodide
SEM: Standard error of the mean
SNAT: Sodium-coupled neutral amino acid transporter
TCA: Tricarboxylic acid
TCCLE: The Cancer Cell Line Encyclopedia
tRNA: Transfer RNA
