# Supplementary material for: Benzylserine inhibits breast cancer cell growth by disrupting intracellular amino acid homeostasis and triggering amino acid response pathways
Source: BMC Cancer. 2018 Jun 26;18:689. doi: 10.1186/s12885-018-4599-8 (PMC6019833; doi:10.1186/s12885-018-4599-8)
Supplement: Supplementary file 1 — Table S1. Summary of [3H]-L-glutamine and [3H]-L-leucine uptake IC50 values for breast cancer cells treated with varying concentrations of BenSer. Table S2. Summary of Kruskal-Wallis tests with Dunn’s multiple comparisons correction on inter-subtype comparisons of gene expression (mRNA log2 values) of putative BenSer targets SLC7A5, SLC3A2, SLC7A8, SLC1A5, SLC1A4, SLC38A1, and SLC38A2 in the METABRIC dataset (n = 2509). Data were grouped into the “PAM50 + Claudin-low” subtypes based on clinical attribute data retrieved from www.cbioportal.org/. *p < 0.05, **p < 0.01, ***p < 0.001, ****p < 0.0001, ns = not significant. (PDF 308 kb) [file 12885_2018_4599_MOESM1_ESM.pdf]

Table S1. Summary of [^{3}H]-L-glutamine and [^{3}H]-L-leucine uptake IC_{50} values for breast cancer cells treated with varying concentrations of BenSer.

	**[^{3}H]-L-glutamine IC_{50}**				**[^{3}H]-L-leucine IC_{50}**			
Cell line	MCF-7	HCC1806	MDA-231	MCF10a	MCF-7	HCC1806	MDA-231	MCF10a
IC_{50}	37.85	17.19	15.37	3.901	3.789	2.87	3.001	17.63
Span (%)	= 100	= 91.81	= 80.01	= 65.15	= 93.74	= 97.71	= 98.58	= 100

Supplementary Table 2. Summary of Kruskal-Wallis tests with Dunn's multiple comparisons correction on inter-subtype comparisons of gene expression (mRNA log_2 values) of putative BenSer targets SLC7A5, SLC3A2, SLC7A8, SLC1A5, SLC1A4, SLC38A1, and SLC38A2 in the METABRIC dataset (n=2509). Data were grouped into the "PAM50 + Claudin-low" subtypes based on clinical attribute data retrieved from www.cbioportal.org/. *p < 0.05, **p < 0.01, ***p < 0.001, ****p<0.0001, ns=not significant.

	**Gene**								
**Subtype comparison pairs**	***SLC7A5***	***SLC3A2***	***SLC7A8***	***SLC43A1***	***SLC43A2***	***SLC1A4***	***SLC1A5***	***SLC38A1***	***SLC38A2***
Basal vs. HER2	****	ns	****	ns	**	****	**	****	****
Basal vs. Luminal A	****	***	****	****	****	****	****	****	ns
Basal vs. Luminal B	****	ns	****	*	****	****	ns	****	**
Basal vs. Normal	****	ns	****	**	***	****	****	****	*
Claudin-low vs. HER2	***	ns	****	ns	****	****	****	****	*
Claudin-low vs. Luminal A	****	***	****	****	****	****	ns	****	ns
Claudin-low vs. Luminal B	**	ns	****	ns	****	****	****	****	ns
Claudin-low vs. Normal	***	ns	****	*	****	****	ns	****	ns
HER2 vs. Luminal A	****	ns	****	**	ns	ns	****	ns	****
HER2 vs. Luminal B	****	**	****	ns	****	ns	****	ns	****
HER2 vs. Normal	****	ns	ns	ns	ns	ns	****	ns	*
Luminal A vs. Luminal B	****	****	***	ns	ns	ns	****	ns	ns
Luminal A vs. Normal	***	ns	***	ns	ns	ns	ns	ns	ns
Luminal B vs. Normal	ns	ns	ns	ns	*	ns	****	ns	ns
