# Supplementary figures and images for: Benzylserine inhibits breast cancer cell growth by disrupting intracellular amino acid homeostasis and triggering amino acid response pathways
Source: BMC Cancer. 2018 Jun 26;18:689. doi: 10.1186/s12885-018-4599-8 (PMC6019833; doi:10.1186/s12885-018-4599-8)

# Figure S1

## A

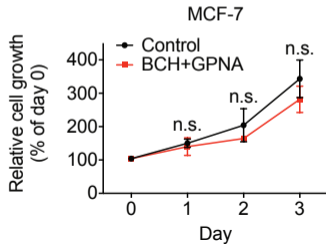

## B

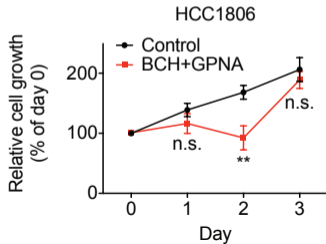

# Supplementary Figure 2

## A

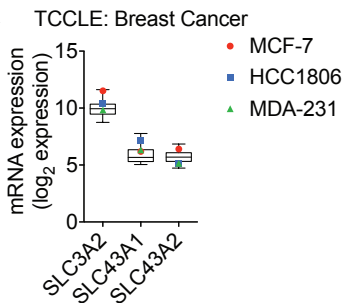

## B

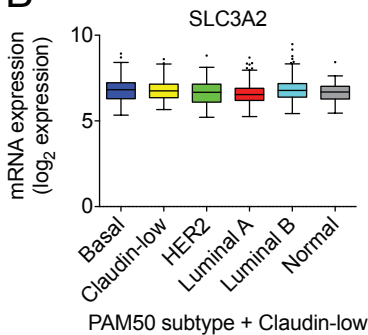

## C

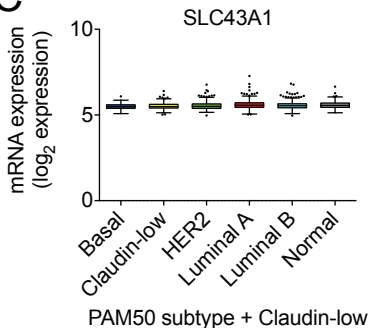

## D

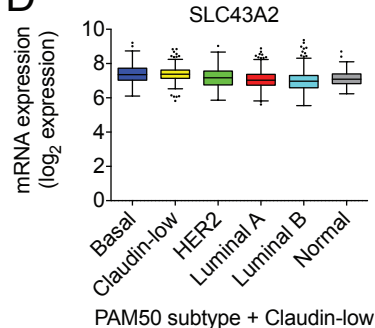

# Supplementary Figure 3

A

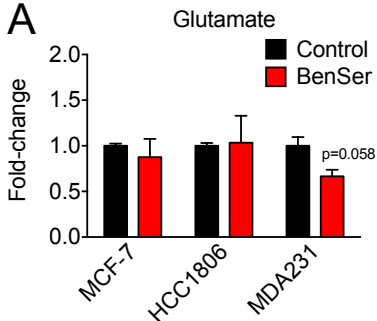

B

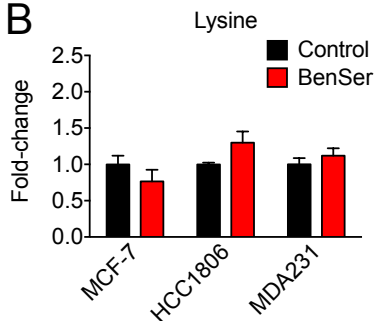

C

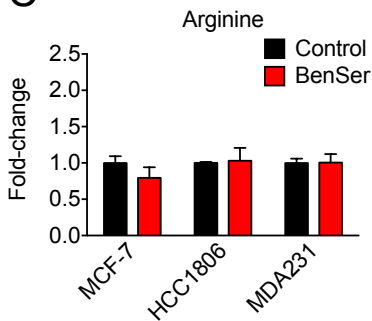

D

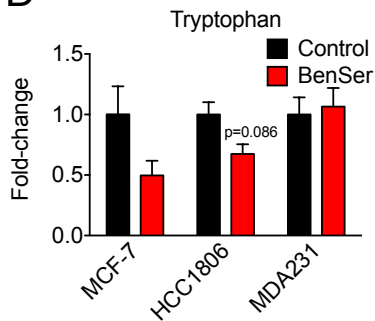

# Supplementary Figure 4

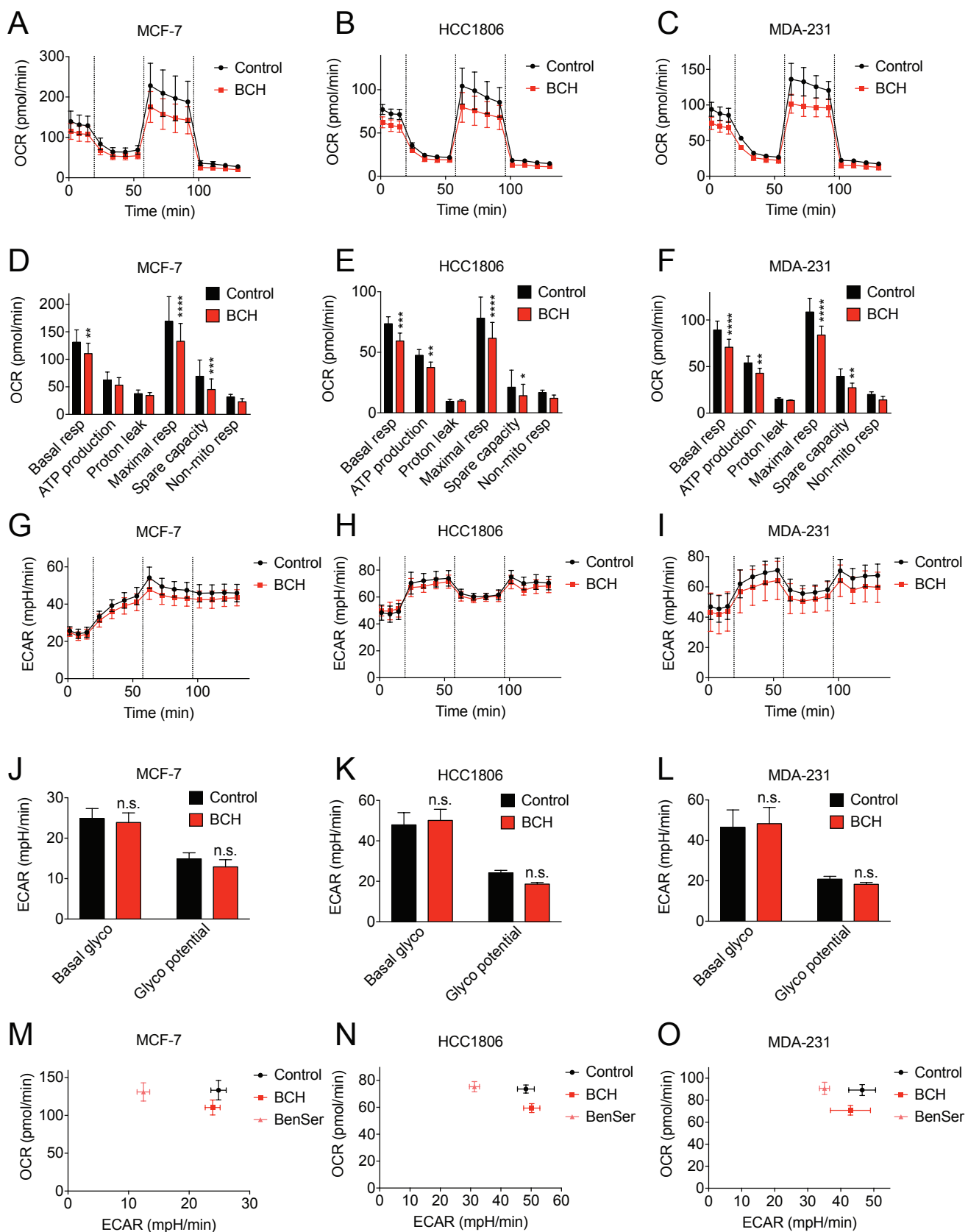

Supplement: Supplementary file 2 — Figure S1. Combined LAT1 and ASCT2 inhibition does not recapitulate the effects of BenSer. Relative cell viability measured by MTT assay in MCF-7 (A) and HCC1806 (B) cells cultured for 3 days in the presence or absence of 10 mM BCH and 1 mM GPNA. Data represent mean ± SEM of at least three independent experiments. *p < 0.05, **p < 0.01, ***p < 0.001, ****p < 0.0001; 2-way ANOVA. Figure S2. Expression of putative BenSer targets in breast cancer cell lines and patient samples. A, gene expression (mRNA log2 values) of LAT common heavy chain (SLC3A2) and LAT transporters (LAT3/SLC43A1, LAT4/SLC43A2), was analysed in all breast cancer cell lines (n = 55) included in The Cancer Cell Line Encyclopedia (TCCLE). Grouped data are plotted as box-and-whisker plots (max to min), with log2 mRNA expression in MCF-7 (red), HCC1806 (blue) and MDA-MB-231 (MDA-231) cells overlaid as individual data points. B, mRNA expression (log2 values) of SLC3A2 (B), SLC43A1 (C) and SLC43A2 (D), in the METABRIC dataset (n = 2509). Data were grouped into the “PAM50 + Claudin-low” subtypes based on clinical attribute data retrieved from www.cbioportal.org/ and are plotted as box-and-whisker plots (Tukey). Figure S3. Intracellular levels of amino acids that are not affected by BenSer treatment. Intracellular levels of glutamate (A), lysine (B), arginine (C), and tryptophan (D) measured in MCF-7, HCC1806 and MDA-MB-231 (MDA231) cells after 14 h incubation in the presence or absence of 10 mM BenSer. Data are normalised to cellular protein content and expressed as a fold-change compared to Control. Data represent mean ± SEM of two independent experiments performed in triplicate. For (D), p-value shown was calculated with unpaired student’s t-test. Figure S4. BCH treatment reduces OCR. Oxygen consumption rate (OCR; A-F) and extracellular acidification rate (ECAR; G-L) in MCF-7, HCC1806 and MDA-MB-231 cells treated in the presence or absence of 10 mM BCH were assessed using a Seahorse Mito Stress Test. Dat [file 12885_2018_4599_MOESM2_ESM.pdf]
